# Recognition of Conserved Amino Acid Motifs of Common Viruses and Its Role in Autoimmunity

**DOI:** 10.1371/journal.ppat.0010041

**Published:** 2005-12-16

**Authors:** Mireia Sospedra, Yingdong Zhao, Harald zur Hausen, Paolo A Muraro, Christa Hamashin, Ethel-Michele de Villiers, Clemencia Pinilla, Roland Martin

**Affiliations:** 1 Cellular Immunology Section, Neuroimmunology Branch, National Institute of Neurological Disorders and Stroke, National Institutes of Health, Bethesda, Maryland, United States of America; 2 Computational and System Biology Group, Biometric Research Branch, National Cancer Institute, National Institutes of Health, Bethesda, Maryland, United States of America; 3 Division for the Characterization of Tumorviruses, Deutsches Krebsforschungszentrum (German Cancer Research Center), Heidelberg, Germany; 4 Mixture Sciences, San Diego, California, United States of America; 5 Torrey Pines Institute for Molecular Studies and Mixture Sciences, San Diego, California, United States of America; Washington University School of Medicine, United States of America

## Abstract

The triggers of autoimmune diseases such as multiple sclerosis (MS) remain elusive. Epidemiological studies suggest that common pathogens can exacerbate and also induce MS, but it has been difficult to pinpoint individual organisms. Here we demonstrate that in vivo clonally expanded CD4^+^ T cells isolated from the cerebrospinal fluid of a MS patient during disease exacerbation respond to a poly-arginine motif of the nonpathogenic and ubiquitous Torque Teno virus. These T cell clones also can be stimulated by arginine-enriched protein domains from other common viruses and recognize multiple autoantigens. Our data suggest that repeated infections with common pathogenic and even nonpathogenic viruses could expand T cells specific for conserved protein domains that are able to cross-react with tissue-derived and ubiquitous autoantigens.

## Synopsis

Infectious agents have been discussed as possible triggers for multiple sclerosis (MS). Molecular mimicry, meaning an antigenic similarity between pathogen proteins and self-proteins (also called autoantigens), is one mechanism that can activate autoreactive T cells. To identify potential triggers and autoantigens in MS, the authors of this study determined the specificity of T cell clones (TCCs) from cerebrospinal fluid (CSF) of an MS patient, which were clonally expanded during disease exacerbation. The CSF is in intimate contact with the central nervous system, which is damaged by autoreactive T cells in MS. The authors observed that these TCCs recognize amino acid motifs from functional protein domains that are evolutionarily conserved between viruses, prokaryotes, and eukaryotes. This phenomenon is reminiscent of pattern recognition by the innate immune system via Toll-like receptors, and represents an interesting bridge as to how immune responses against foreign agents may be misdirected against autoantigens. Three TCCs recognize arginine-rich motifs and respond to peptides from the ubiquitous, nonpathogenic Torque Teno virus (TTV), but also from other common viruses and autoantigens. TTV recognition by clonally expanded CSF TCCs, and the demonstration of viral infection in brains of people with MS, suggest that this virus may participate in triggering or sustaining autoimmune diseases such as MS.

## Introduction

Multiple sclerosis (MS) is considered a CD4^+^ T helper-1-mediated autoimmune disease that affects the central nervous system (CNS). The etiology of MS remains unclear, but the disease develops in genetically susceptible individuals and likely requires environmental triggers. Epidemiological studies have shown that MS relapses often follow common viral infections, and the viral etiology of certain human demyelinating diseases, and studies of virus-induced disease models [1–3], all point to a role of viruses in MS. Numerous agents have been linked with MS based on serology, pathology, or virus isolation, but none of the associations has been conclusive. The difficulty in identifying a single microorganism as the cause of MS or other autoimmune diseases could indicate that Koch's paradigm, “one organism - one disease,” does not apply to such complex diseases, although we should not discard the possibility that “the” MS agent may still be discovered. However, the bulk of current data suggests that MS is induced and/or exacerbated by several different agents, and that these are most likely ubiquitous pathogens and highly prevalent in the population.

Molecular mimicry, i.e., cross-recognition of foreign agents and self-proteins, is one mechanism by which infectious agents can induce autoimmune diseases [4]. In this context, the traditional search for triggers for MS has been based on choosing a likely target autoantigen (e.g., myelin), establishing CD4^+^ T cells specific for immunodominant peptides, and then searching for molecular mimics in viral and bacterial databases [5]. The main drawbacks of previous studies are that (a) autoreactive T cells have been established almost universally from the peripheral blood instead of an affected tissue compartment and without knowledge about whether they are related to disease activity; and (b) they have been established based on the consideration that some or all autoreactive T cells in MS are reactive to myelin [6]. This last reasoning is probably too simplistic, since autoimmune diseases can be exquisitely organ- or tissue-specific, and nevertheless autoimmune T cells can be directed against ubiquitous autoantigens, e.g., pyruvate dehydrogenase in primary biliary cirrhosis [7]. To overcome the above problems, we decided to isolate T cells from the cerebrospinal fluid (CSF), a compartment in intimate contact with the affected brain tissue, and focus on T cells that are clonally expanded in vivo during active disease, and hence likely relevant to the autoimmune disease process. Our methodological approach combines unbiased expansion of virtually every T cell using a universal T cell stimulus (phytohemagglutinin [PHA]) [8] with determination of in vivo clonal expansion by T cell receptor (TCR) complementarity-determining region 3 (CDR3) spectratyping, and the unbiased identification of stimulatory peptides by integration of data from screening of positional scanning synthetic combinatorial peptide libraries (PS-SCLs) with protein database analyses [9,10].

## Results

### Generation of In Vivo-Expanded, CSF-Infiltrating T Cell Clones

**Figure 1:**
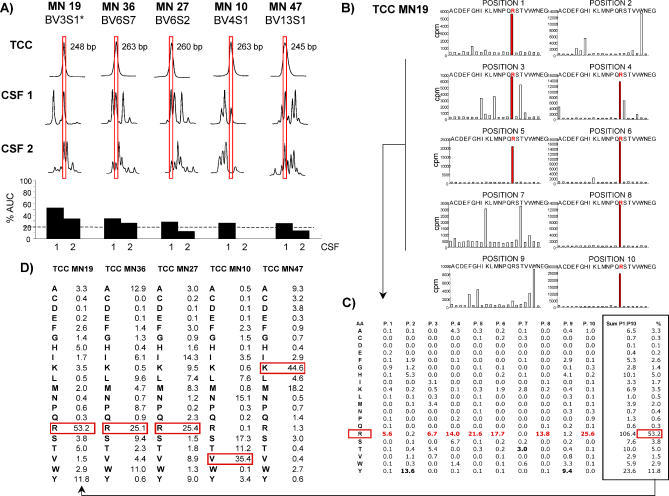
In Vivo-Expanded CSF-Infiltrating TCCs and Their Response to PS-SCL (A) TCR BV rearrangement (*Arden's nomenclature) of selected TCCs. Histograms of the relative CDR3 length distributions of each TCC (top histogram) and CSF T cells (middle and bottom histograms). Fluorescence intensity is listed on the y-axis, and on the x-axis, the electrophoresis time resolving in-frame rearrangements of TCRB CDR3 at 3-nt intervals. Red boxes identify the correct alignment. The bottom graph represents the percent contribution (expressed as AUC) of the TCCs' CDR3 to all CDR3s with the same BV chain in the CSF samples. (B) Proliferative response of MN19 to a complete decapeptide PS-SCL. Single-letter aa codes are listed on the x-axes, and proliferation (cpm) is shown on the y-axis. Data represent one experiment of three. The mixtures with R as the defined aa inducing the highest response are shown in red. (C) Score matrix for MN19. Each number represents the SI_PS-SCL_ (mean of three independent experiments) of each of the 200 mixtures of a decapeptide PS-SCL (rows, aa; columns, positions). The last column represents the optimal composition of stimulatory peptides “peptidome.” The aa contributing the most to “peptidome” is shown in red. (D) Peptidomes of the five in vivo-expanded TCCs.

We isolated CSF-infiltrating cells from a patient with relapsing-remitting MS during disease exacerbation and cloned them by limiting dilution with PHA as an unbiased stimulus [11]. Growing colonies were characterized for CD4/CD8 and TCRVβ expression, and clonality was confirmed by TCR-variable (TCR *VB*) chain sequencing (Figure 1A and Table 1). In vivo clonal expansion was assessed by TCR CDR3 spectratyping [12], by comparing the CDR3 length of individual T cell clones (TCCs) with the CDR3 spectrum of CSF mononuclear cells at the time of the lumbar puncture during disease exacerbation (CSF1), and again 14 mo later (CSF2), during remission. Our analysis focused on five CD4^+^ TCCs (MN10, MN19, MN27, MN36, and MN47), because they were clonally expanded in vivo at exacerbation and reduced 14 mo later, suggesting involvement in disease exacerbation (Figure 1A). Each of these TCCs produced T helper-1 cytokines, and their HLA-class II restriction was also characterized (Table 1).

**Table 1:**
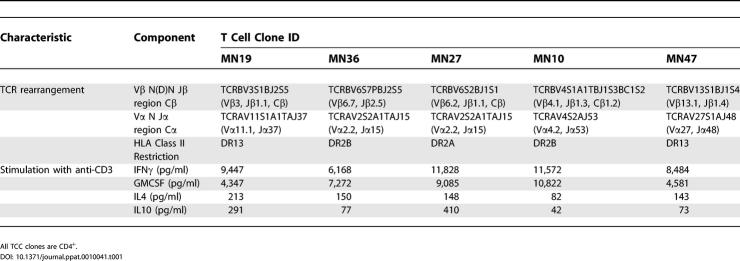
Characterization of TCCs

### CD4^+^ T Cell Clones Show Preferential Recognition of Specific Amino Acids

Subsequently, we tested the five TCC with a decapeptide PS-SCL as described [9,10], which allows the identification of stimulatory peptides. The results of a representative experiment for TCC MN19 are presented in Figure 1B (for the other four TCC, see Figure S1). Most remarkably, each of the five TCCs responded strongest to one defined amino acid (aa) in multiple positions of the libraries (marked in red in Figures 1B and S1). Based on the stimulatory indices (SIs) from testing the PS-SCL mixtures (SI_PS-SCL_), we generated a scoring matrix for each TCC as described [10]. The matrix for MN19 is shown in Figure 1C. We used the matrix to estimate for each TCC the preference of a given aa in the composition of the predicted stimulatory peptides (“peptidome”) by calculating the sum of the SI_PS-SCL_ in each of the ten positions for each of the 20 L-amino acids (L-aa) and expressing it as a fraction of 100% (Figure 1C). If we assume a uniform distribution of the 20 aa in each position, the probability of each aa in each position would be 5%. The “peptidome,” or composition of the predicted stimulatory peptides, for each TCC shows a substantial preference for one aa (Figure 1D, boxed in red): in three TCCs the preference is for R (MN19 [53.2%], MN27 [25.4%], and MN36 [25.1%]), in one TCC for V (MN10 [35.4%]), and in one TCC for K (MN47 [44.6%]).

### CSF-Infiltrating T Cell Clones Recognize Torque Teno Virus

Next, we predicted stimulatory peptides for these CD4^+^ TCCs by PS-SCL biometrical analysis [10], and then selected peptides from human infectious agents (see Materials and Methods). Table 2 shows the actual number of predicted bacterial and viral peptides (column labeled #) and this number normalized for the total number of decamer entries in the database multiplied by one million to facilitate analysis (column labeled NPP). The normalization step was performed to avoid biased representation of agents with large numbers of sequence entries such as HIV or *Escherichia coli*.

**Table 2:**
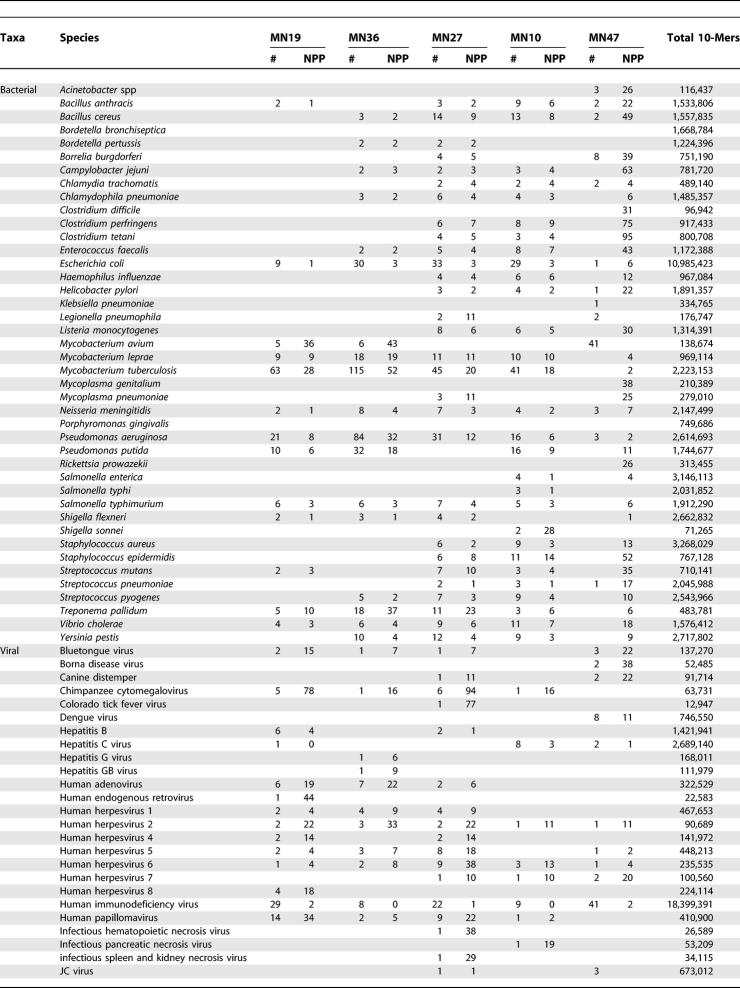
Human Infectious Agents Predicted to Be Recognized by the TCCs

**Table 2:**
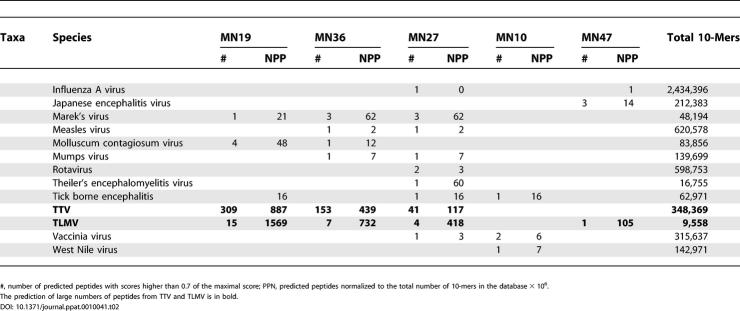
Continued

Although the prediction included a few peptides from bacteria that had previously been related to MS or CNS infections, the NPP values for these agents were very low. Furthermore, no specific bacterium was preferentially recognized by the three TCCs with R-enriched peptidomes (Table 2). In contrast, and exclusively for these three TCCs, much higher values (NPP > 100) were found for two related small DNA viruses, Torque Teno virus (TTV) and TTV-like mini virus (TLMV) (Table 2 in bold). TTV was identified in 1997 [13] and thought to mediate hepatitis infection, although further follow-up did not confirm this suspicion [14,15]. TLMV was identified in 1999 in a study of TTV prevalence in blood donors [16]. These two viruses are now recognized as ubiquitous agents in the human population and are considered orphan viruses, i.e., they have not been related to a known disease. Interestingly, in the context of MS, TTV DNA has been detected in CSF and brain samples [17,18]. We have confirmed this neurotropism by detecting TTV DNA in five of 11 brain samples from MS patients (Tables 3 and S1) and also in 32 of 41 brain tumors (astrocytoma, medulloblastoma, and ependymoma) (E. M. de Villiers and W. Scheurlen, unpublished data). Interestingly, an identical sequence was detected in three different patients.

**Table 3:**
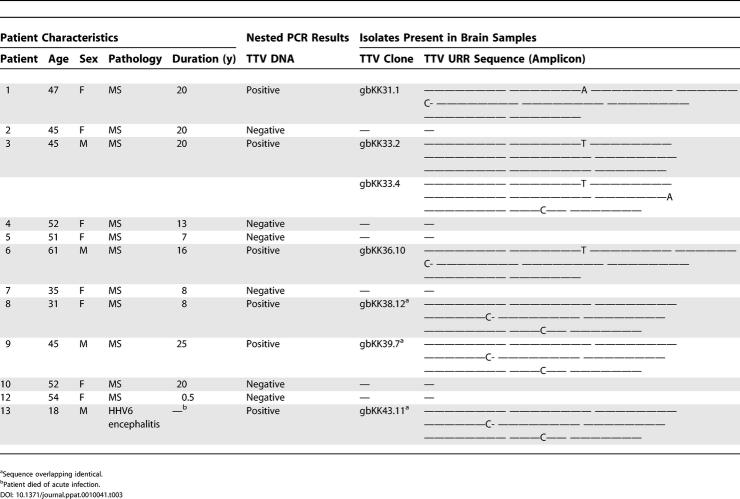
Detection of TTV-DNA in MS Brain

**Figure 2:**
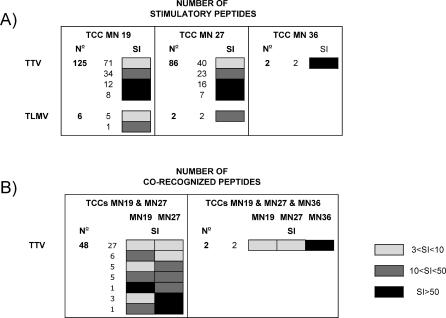
Stimulatory Peptides from TTV and TLMV (A) Number and SIs of the stimulatory peptides from TTV and TLMV identified for TCCs MN19, MN27, and MN36. (B) Number and SIs of the peptides co-recognized by different TCCs. Peptides have been tested in proliferation assays at 10 μg/ml and using PBMCs as antigen-presenting cells.

Next, we tested a total of 228 TTV and TLMV peptides predicted to be recognized by MN19, MN27, or MN36 with higher scores, particularly those with scores higher than 0.7 of the maximal theoretical score. Assuming that the shared preference for R-enriched peptides by these three TCCs reflects similarities in specificity, we tested the peptides predicted for one TCC also with the other two TCCs. The number of stimulatory peptides identified for each TCC is summarized in Figure 2A. Detailed information about all stimulatory peptides is available in Table S2. Fewer peptides are stimulatory for MN36, although in contrast to MN19 and MN27, MN36 preferentially recognizes peptides longer than decamers. Since the current search strategy is based on decamer libraries, our prediction efficacy was therefore much lower for MN36. Despite this, it is interesting to note that all three TCCs recognize peptides not only from one organism, TTV, but even the same peptides. The few TTV peptides that stimulate MN36 are recognized by all three TCCs, and 48 peptides are recognized by MN19 and MN27 (Figure 2B). However, as shown in Figure S2 by dose titration experiments, the different clones recognize these peptides with different affinities, i.e., at different concentrations.

### MS Patient Is Infected with TTV at Disease Exacerbation

**Figure 3:**
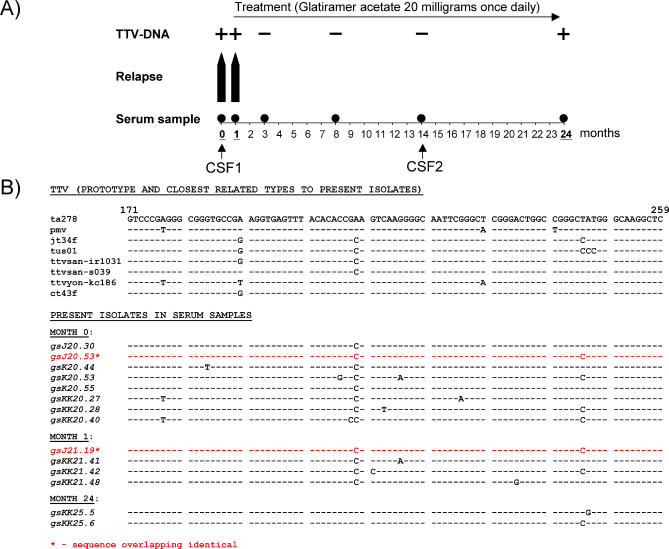
TTV-DNA in Patient's Serum Samples (A) Detection of TTV-DNA by PCR amplification using two primer combinations with the respective nested primers. Six different serum samples from the same patient obtained at different time points were analyzed. The first two samples were obtained during relapse. Time points with simultaneous CSF are indicated. Plus symbol indicates presence of TTV DNA; minus symbol indicates absence. (B) The sequences obtained by cloning and sequencing of all amplicons and the alignment with the closer related known TT viruses are shown. One isolate that was present in two different serum samples is shown in red.

In order to determine whether the MS patient was infected with TTV, serum samples from the time of the first CSF isolation (time 0) and 1, 3, 8, 14, and 24 mo later, were tested for TTV-DNA by PCR amplification using two primer combinations with the respective nested primers. Cloning and sequencing of all amplicons confirmed the presence of TTV DNA in the first serum sample obtained during exacerbation and also 1 mo later, during a second exacerbation (Figure 3A and Table S1). The next three serial samples during remission were negative, but TTV-DNA was again detected 24 mo later, suggesting a second infection or reactivation. The highly conserved region of the TTV upstream regulatory region (URR) amplified here did not allow for determination of the specific TTV types involved. However, sequence alignment of the samples with the closest related known TTV strains indicates that different isolates were present in the positive serum samples (Figure 3B). Seven different isolates were identified at month 0, three at month 1, and two at month 24; and only one isolate was shared by two isolates (time 0 and 1 mo). Interestingly, this isolate was identical to the one shared by the three brain samples (see above). The CSF could not be checked for TTV infection, as CSF became unavailable.

The absence of TTV-DNA in the serum during remission does not exclude its persistence in infected cells, since presence of TTV DNA in tissue samples has been reported despite its absence in corresponding sera [17].

### Cross-Recognition of Conserved TTV Epitopes

**Figure 4:**
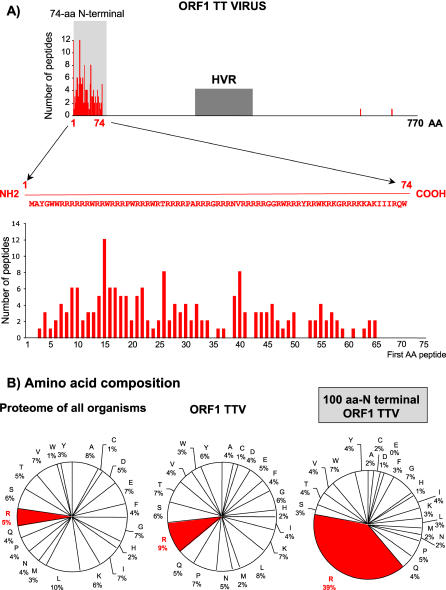
Characterization TTV Peptides (A) Configuration of ORF1 from TTV showing the hypervariable region (HVR) and the 74-aa N-terminal domain. Sequence of the first 74 aa for a prototype TTV is shown. Distribution of all TTV stimulatory peptides identified between the first 74 aa is shown. (B) AA composition of all proteins, ORF1 from TTV, and the 74-aa N-terminal region of ORF1.

Next we examined the location of each individual stimulatory peptide (Table S2). TTV has five open reading frames (ORFs) and, interestingly, 96.2% (152/158) of the stimulatory peptides were from a narrow region in the 74-aa N-terminal sequence of ORF1 (Figure 4A). ORF1 and especially its N-terminal region are highly enriched in positively charged aa (mainly R) when compared to the proteomes of all organisms (Figure 4B). The N-terminal R-rich domain of TTV ORF1 corresponds to a potential nuclear localization signal (NLS). A significant proportion of the stimulatory peptides identified are shared by different TTV isolates, indicating that this N-terminal sequence is conserved (Table S2).

**Figure 5:**
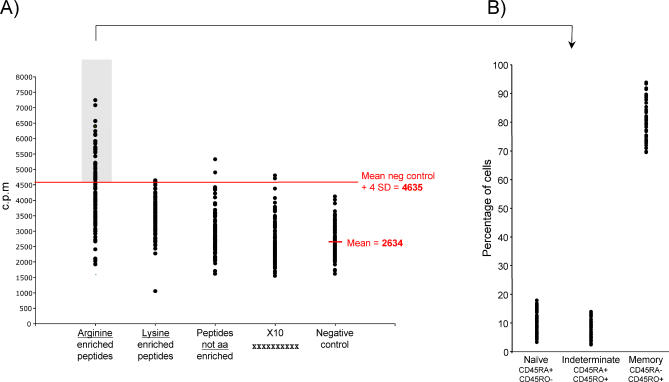
TCL Response to R-Enriched Peptides in Peripheral Blood (A) Proliferative response of peripheral TCL to the following: a mixture of ten peptides enriched in R, a mixture of ten peptides enriched in K, a mixture of ten peptides not enriched in any specific aa, and a mixture of randomized decapeptides. Negative control is medium without peptide mixture. Responses higher that the mean of the negative control plus 4 standard deviations have been considered positive. * Detailed information of mixtures used in IL-7 primary proliferation assay are available in Table S3. (B) Comparison of the origin (naïve versus memory) of all TCLs with confirmed reactivity against R-enriched peptides.

Since multigenotype and persistent infections with TTV are frequent, an expansion of TCCs recognizing these conserved domains is expected. We therefore addressed whether the precursor frequency of T cells responding to R-enriched peptides was increased in the patient in our study. Using IL-7 primary proliferation assays, we stimulated peripheral blood cells with mixtures of ten peptides enriched in R, ten peptides enriched in K, ten peptides not enriched in any specific aa, and a decamer peptide mixture in which all 20 L-aa are present in randomized order in each of the ten positions (X10). Detailed information about these mixtures is available in Table S3. We demonstrated a higher frequency of peripheral T cells specific for TTV R-enriched peptides up to 3 y after the last exacerbation (Figure 5A). The majority of these T cells originated from the memory T cell pool (Figure 5B).

### Cross-Recognition of Conserved R-Rich Protein Domains in Common Viruses

We also identified stimulatory peptides for these three TCCs from other common human viruses, particularly adenovirus and papillomavirus (Table 4). Interestingly, and similar to what we observed for TTV, various peptides are located in the same protein region and shared by different, but related, strains of one virus. The four stimulatory peptides identified from different types of human papillomavirus are located between aa 447–461 in the minor capsid protein L2 (Table 4). The C terminus of this protein is enriched in basic aa, and the region between aa 456 and 461 is described as a putative NLS [19]. Regarding human adenovirus, we identified three different stimulatory peptides from pVII protein shared by different strains (Table 4). PVII protein is a core protein enriched in R with a histone-like function and tightly bound to the viral DNA. Previous observations indicate that this close association is likely preserved during transport of the viral genome to the nucleus [20]. Interestingly, both proteins share important characteristics with the 74-aa N-terminal sequence of ORF1 from TTV.

**Table 4:**
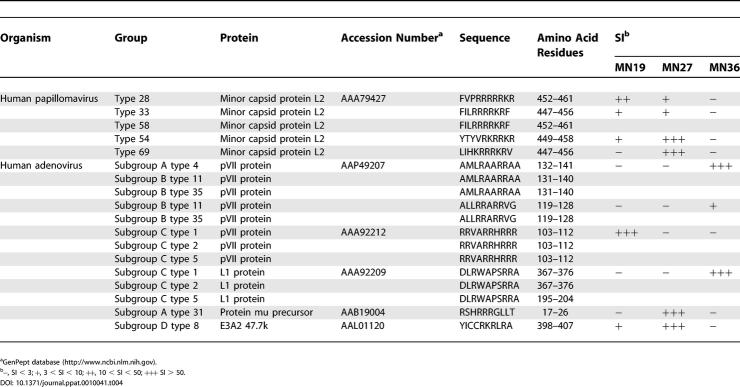
Stimulatory Peptides from Common Viruses

### TTV-Specific T Cells Cross-React with R-Rich Domains in Autoantigens

R-enriched domains are frequent in eukaryotes, prokaryotes, and viruses as part of DNA-binding regions, NLSs, and other functional sequences, providing a bridge for cross-reactivity between pathogens and autoantigens. In support of this notion, three of the stimulatory TTV peptides recognized by TCC MN19 are identical to peptides from two human proteins (Table 5). The first peptide stems from a DEAD box protein. Although the function of most DEAD box proteins is unknown, helicase activity and interactions with DNA or RNA have been associated with several of them [21]. The other two peptides are part of the α-1B adrenergic receptor (α_1_-AR). They are located between aa 367–380, an R-rich motif that has been reported to interact with the multifunctional protein gC1qR, controlling their expression and subcellular localization [22].

**Table 5:**
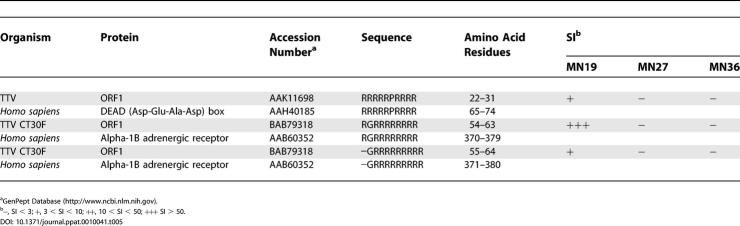
Peptides Derived from TTV and Human Proteins with Identical Peptide Sequences

TCC MN19 is the most expanded clone, and it shows the highest preference for R-enriched peptides and, consequently, for R-rich protein domains. For this TCC we have identified five overlapping decapeptides from α_1_-AR (Table 6), two of these identical to two TTV peptides, that completely cover the R-rich motif between aa 367–380 (see above). MN19 also cross-recognizes two peptides from other adrenergic receptors and three overlapping peptides from the N-terminal R-enriched region of ARP (i.e., arginine-rich protein). Although the expression and function of this protein are unknown, starting at aa 56 the translated DNA shares 100% sequence identity with the secreted human protein MANF (mesencephalic astrocyte-derived neurotrophic factor), which protects dopaminergic neurons in the substantia nigra of the brain [23].

TCCs MN27 and MN36 showed a lower preference for R-enriched peptides than did MN19, which is reflected in the corresponding “peptidomes” and in the composition of the stimulatory peptides. Despite this relatively lower recognition of R-rich domains, we nevertheless identified several stimulatory decapeptides with at least three arginines and potential biological relevance in the context of the CNS or the immune system (Table 6). Among these CNS-related molecules is the purinergic receptor PX2A, which is expressed on oligodendrocytes and astrocyte; several other neurotransmitter receptors; ion channels; and molecules that play a role in brain function and metabolism. Among the immunologically relevant autoantigens stimulatory for MN27 and MN36 are immunoglobulin chains and the pattern recognition receptor TLR9. These antigens are of particular interest in the context of perpetuating the autoimmune response once it has started in the CNS.

**Table 6:**
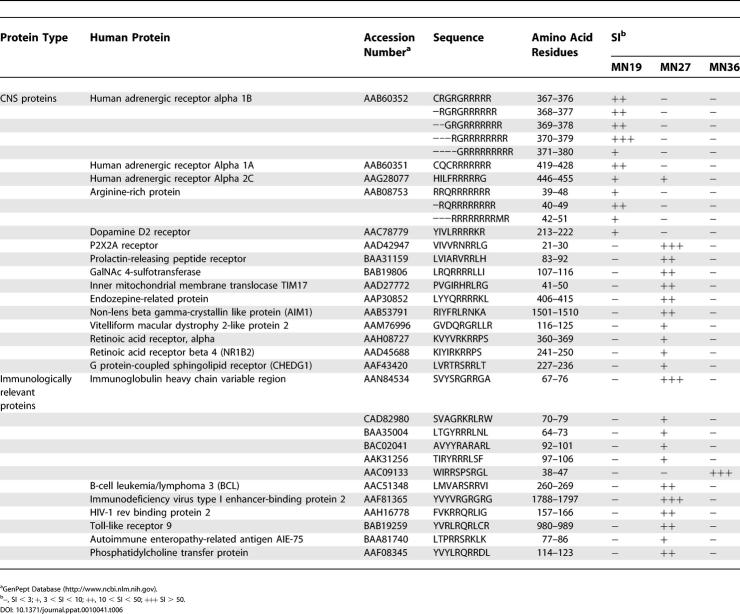
Cross-Reactive Autoantigens

## Discussion

In order to identify putative triggers in MS, we focused in the current study on TCCs that are clonally expanded in vivo at the time of disease exacerbation and in a tissue that is in intimate contact with the affected CNS. Taking T cells directly from the brain or reinjecting potentially autoreactive T cells back into a patient would provide more direct evidence for the relation to disease, but for obvious reasons these steps are either very difficult to justify, i.e., brain biopsies for nondiagnostic reasons, or impossible in humans. Thus, as a next step, we applied methods that included the unbiased expansion of T cells and a search strategy using combinatorial peptide chemistry and bioinformatics. This approach allowed, to our knowledge for the first time, the identification of target epitopes of T cells for which nothing was known in terms of their antigen specificity a priori.

The first interesting and novel point of our data is that the recognition pattern of the five TCCs we studied showed a preference for one specific aa at several positions of the PS-SCL that translates into recognition of peptides enriched in this aa. The most stimulatory peptides for MN19, which shows the strongest bias for R (53.2%), contain on average 7.8 R residues within a decamer. The preference for R-enriched peptides shared by the clones MN19, MN27, and MN36, together with their marked clonal expansion in the CSF, suggest that these three TCCs could have been activated in the periphery in response to the same foreign agent(s) before migrating to the CSF. The analysis of predicted peptides from human infectious agents confirmed this hypothesis. A large number of peptides from TTV and TMLV were predicted exclusively for the three TCCs with the preference for R-enriched peptides. At this stage, no other pathogen was predicted at a comparable level. We therefore assumed that TTV and related viruses are the most likely candidate targets for MN19, MN27, and MN36. We synthesized a large number of these predicted peptides and found not only multiple stimulatory TTV peptides for each of the three TCCs, but many that were recognized by two or even all three TCCs. With few exceptions all peptides are located in an R-rich area of TTV ORF1, a putative capsid protein that is thought to mediate binding to viral DNA and transport to the nucleus. The chromatin association of this R-enriched region of TTV has been demonstrated in in vitro experiments (R. Kellner and E. M. de Villiers, unpublished data). This region is shared by different TTV isolates, suggesting that it represents a conserved functional domain. Human T cell recognition of epitopes conserved between different but related subtypes of viruses has been described for enteroviruses [24,25], flaviviruses [26], influenza A virus [27], and adenoviruses [28]. It has been proposed that exposure to consecutive infections with different strains results in repeated cycles of stimulation and expansion of T cells specific for shared epitopes [27]. The frequent multigenotype infections that characterize TTV and occurred in the patient in this study, together with the unusually large size of the R-rich domain (74 aa) in this virus, are likely relevant in amplifying T cell expansion, a notion supported by the high frequency of peripheral memory T cells specific for R-enriched peptides.

The fact that basic aa such as R play an important role in the interaction of proteins with DNA and with other proteins such as shuttle proteins implies that R-enriched domains are frequent in all organisms as part of DNA/RNA-binding regions, NLSs, or other functional domains. The recognition of such evolutionarily conserved domains by adaptive immune cells such as the TCCs examined here is reminiscent of pattern recognition by innate immune receptors such as Toll-like receptors and could facilitate the cross-recognition of different organisms and human proteins. The fact that several stimulatory peptides from common human viruses other than TTV, such as adenovirus and papillomavirus, are also from R-enriched protein domains with characteristics similar to the 74-aa N-terminal region of ORF1 supports this hypothesis. Important additional evidence includes the identity between three stimulatory TTV peptides and three peptides from human proteins with functional R-enriched domains. A series of other interesting R-enriched peptides from autoantigens have been identified, including several overlapping peptides from a R-rich motif of α_1_-AR. The density of α_1_-ARs in the CNS is among the highest of any tissue in the body [29], and although the specific functional roles of this receptor remain uncertain, it has been implicated in motor control by the CNS [30–33]. Interestingly, no myelin autoantigens were among the autoantigenic peptides with highest predicted stimulatory scores for these TCCs. This could be explained by the fact that axonal damage, gliosis, and inflammation also play a role besides demyelination, and that nonmyelin autoantigens, such as alpha-B crystalline, S-100, and others, have already been implicated in MS or experimental allergic encephalomyelitis.

One important component that remains unidentified is the initial activator of these T cells. Both TTV infections and autoantigens are plausible. However, since TTV infections occur frequently and probably also early in life, and since activation of T cells probably starts in the periphery and transmigration into the CNS/CSF and damage of tissue are subsequent events, we believe that the most likely scenario may be that repetitive TTV infections lead to expansion of these T cells.

T cell recognition of R-enriched peptides may not be critical for clearance of the infectious agent, since most of these peptides are recognized at only moderate concentrations, suggesting low functional avidity of the T cells. Furthermore, deletion of high-avidity cells specific to these common, conserved domains in the thymus is expected. However, the frequent occurrence of these peptides in nature implies that T cells specific for R-enriched areas may be activated repeatedly, resulting in lower requirements for costimulation and expansion despite relatively low functional avidity. The resulting reduction in the activation threshold, together with the repetitive expansion of these cells, may facilitate responses to suboptimal autoantigens in the target tissue acting as an “acquired susceptibility trait” in MS. Since TTV infections are also frequent in normal donors, additional predisposing factors, such as human leukocyte antigen and other susceptibility genes, compromised CNS repair processes, increased tissue vulnerability, or variations in central tolerance, are probably necessary for MS development.

Despite this demonstration that several CSF-infiltrating and in vivo-expanded TCCs from an MS patient in exacerbation recognize large numbers of peptides from TTV, we do not suggest this virus as the latest “MS agent.” The fact that these likely disease-related TCCs recognize R-enriched conserved domains shared between different viruses and human autoantigens suggests that the specificity of these T cells results in recognition of specific types of protein domains rather than a specific organism. This kind of specificity or recognition of evolutionarily conserved domains could be involved in inducing and perpetuating autoreactive T cells. It will be important to examine whether the proposed mechanism applies to other MS patients and to other autoimmune diseases, and whether the recognition of conserved protein domains by adaptive immune cells plays a role during protective immune responses.

## Materials and Methods

### TCCs and antigen-presenting cells.

TCCs were established from CSF of an untreated patient with relapsing-remitting MS during exacerbation by limiting dilution at 0.3 and 3 cells/well with 2 × 10^5^ autologous, irradiated PBMCs and 2.5 μg ml^−1^ of PHA-P (Sigma, St Louis, Missouri, United States) in IMDM containing 100 U ml^−1^ penicillin/streptomycin, 50 μg ml^−1^ gentamicin, 2 mM L-glutamine (BioWhittaker, Gaithersburg, Maryland, United States), and 5% human serum (Gemini Bio-products, Woodland, California, United States). After 24 h, 20 U ml^−1^ of human recombinant IL-2 (hrIL-2, National Cancer Institute, National Institutes of Health, Bethesda, Maryland, United States) were added. Cells were restimulated every 2 wk with 2.5 μg ml^−1^ PHA, 20 U ml^−1^ hrIL-2, and autologous irradiated PBMCs, and hrIL2 was added every 3–4 d.

### RT-PCR and sequencing of TCR rearrangements.

TCC TCR *VB* gene usage was analyzed by PCR using 21 TCRAV and 23 TCRBV family-specific oligonucleotide primers. Nucleotide sequencing of PCR products was performed as described [34]. TCR gene designations are in accord with Arden's nomenclature [35].

### Cytokine production.

TCCs were stimulated with coated anti-CD3 antibody, and supernatants were collected after 48 h from cultures with/without antibody. IFN-γ, GM-CSF, IL-4, and IL-10 levels were determined by ELISA following the manufacturer's protocol (Biosource, Camarillo, California). For TCCs MN19, MN27, and MN36 the cytokine production was confirmed with several stimulatory peptides.

### CDR3 spectratyping.

For high-resolution TCR β-chain CDR3 spectratyping, 2.5 μl of PCR product from each TCR-BV were used as template in a 12.5 μl primer-extension (“runoff”) reaction containing 1.25 μl of 5′FAM-labeled BV primer, 0.25 μl of 10 mM dNTPs, 0.06 μl of Pfu DNA polymerase, 1.25 μl of Pfu reaction buffer, and 7.2 μl H_2_O. After thermal cycling (95 °C for 2 min; followed by ten cycles of 94 °C for 20 s, 55 °C for 45 s, and 72 °C for 45 s; and a final extension of 72 °C for 10 min), 2 μl of runoff product was mixed with loading buffer containing four Cy-5-labeled DNA size markers, heat-treated at 80 °C for 2 min, and run on a 6% polyacrylamide gel on an OpenGene (Visible Genetics, Toronto, Ontario, Canada) sequencer. Electropherograms were analyzed for peak size (bp), peak height, and area under the curve (AUC). The percentage represented by each CDR3 peak in a BV spectrum (corresponding to the representation of clonal populations with a given CDR3 length) was calculated according to the formula %AUC BVn = (AUC BVn/AUC all BV) × 100. TCR CDR1, CDR2, and CDR3 boundaries were defined according to the IMGT [36].

### Peptide combinatorial libraries and individual peptides.

A synthetic N-acetylated, C-amide L-aa decapeptide combinatorial library in a positional scanning format (PS-SCL; 200 mixtures) was prepared as described [37]. Each OX9 mixture consists of 3.2 × 10^11^ (19^9^) different decamer peptides at approximately equimolar concentration. Individual decapeptides were synthesized with a custom multiple peptide synthesizer using solid-phase Fmoc chemistry. The purity and identity of each peptide were characterized by mass spectrometry.

### Proliferative assays.

TCC proliferation responses to PS-SCL mixtures or individual decapeptides were tested by seeding in duplicate 2 × 10^4^ T cells and 1 × 10^5^ irradiated PBMCs (3,000 rad) with or without PS-SCL mixtures or individual decapeptides. Because the specificity of TCCs was unknown, PHA-P stimulation served as positive control. Proliferation was measured by methyl-^3^H-thymidine (Amersham Biosciences, Little Chalfont, United Kingdom) incorporation. The stimulatory index for a PS-SCL mixture (SI_PS-SCL_) with an aa defined at one position was calculated as SI_PS-SCL_ =SI′/mean all SI′ in the library, where SI′ = (mean of duplicate cpm, mixture) − (mean cpm, background). Responses to mixtures were considered positive when SI > 2. The SI for individual peptides was calculated as SI = (mean of duplicate cpm, peptide)/(mean cpm, background). Responses to individual peptides were considered positive when SI > 3, cpm > 1,000, and at least three standard deviations above average background cpm in at least three independent experiments.

### Biometric analysis and database searches.

Responses to PS-SCLs were analyzed as described [9,10]. A positional scoring matrix was generated by assigning a value of the stimulatory potential to each of the 20 defined aa in each of the ten positions. Based on a model of independent contribution of individual aa to peptide antigen recognition, the predicted stimulatory score of a given peptide is the sum of the stimulatory potential of all aa contained in the peptide in each position. Using a web-based search tool [38], the scoring matrix was applied to rank, according to their stimulatory score, of all the naturally overlapping 10-mer peptides in the protein sequences within the GenPept database (version 136) (ftp://ftp.ncifcrf.gov/pub/genpept), and for viral peptides, within RefSeq (http://www.ncbi.nlm.nih.gov/RefSeq). We analyzed viral and bacterial peptides with scores higher than 0.7 of the predicted maximal theoretical score (Smax). The cut-off of 0.7 of Smax is based on prior experience of the sensitivity and accuracy of the approach [10,39,40]. We then selected peptides from human infectious agents, counted the predicted peptides from each individual organism, and normalized the value, taking into account the total number of decamers for each organism in the GenPept database, in order to avoid a bias related to the number of database entries. This problem would have otherwise significantly skewed the data for organisms with large numbers of reported sequences, such as HIV.

### Precursor frequency in peripheral blood.

Primary proliferation assays were performed as described [41]. Briefly, PBMCs were seeded in 96-well plates at 1 × 10^5^ cells/well on day 0 in the presence of antigen and IL-7. A mixture of ten R-enriched peptides was used as antigen. Three different controls were included, a mixture of ten K-enriched peptides, a mixture of ten peptides not enriched in any specific aa, and a decamer peptide mixture in which all 20 L-aa are present in randomized order in each of the 10 positions (X10) without any defined aa. After 7 d, cell cultures were divided in half, and positive wells were identified by comparing the amount of actively proliferating cells in split cultures with the proliferation of PBMCs seeded without antigen. The remaining half of the positive wells were restimulated, and a confirmation assay was performed at days 17–19. Confirmed positive cultures were used to determine the naïve versus memory origin of precursors T cells by flow cytometry using anti-CD45RA and anti-CD45RO antibodies (Pharmingen, BD, Palo Alto, California, United States).

### TTV detection.

Total DNA was extracted from serum samples by the High Pure Viral Nucleic Acid Kit (Roche Diagnostics, Penzberg, Germany). DNA from brain samples was extracted using phenol and chloroform-isoamyl alcohol. PCR amplification of each sample was performed twice. The primer combinations NG133-NG352 with nested NG249-NG351 [42] were used to amplify a 134-bp fragment of the TTV URR. The latter overlaps the highly conserved region of 71 bp, which is amplified by primers NG472-NG352 and nested NG473-NG351 [43] used in the second PCR amplification. All amplicons were cloned, and at least 12 clones per sample sequenced. Sequences were compared to all available TTV sequences.

## Supporting Information

Figure S1 Proliferative Response of TCCs MN36, MN27, MN10, and MN47 to 200 Mixtures of a Decapeptide PS-SCL in Which Each Mixture Has One Defined aa in One Position and the Other Positions Contain All L-aa Except Cysteine Horizontal axes, single-letter aa code; vertical axes, proliferation as counts per minute. Data represent one experiment of three. The defined aa inducing the highest response at several positions for each TCC is shown in red.
(61 KB PDF)

Figure S2 Proliferation of TCCs MN19 and MN27 in Response to Different Concentrations of Five Decapeptides Proliferation is shown as SI. Peptide sequences are listed on top.
(32 KB PDF)

Table S1 Proportion of TTV Clones Identified After Single and Nested PCR (55 KB PDF)

Table S2 Stimulatory Peptides from TTV and TLMV (71 KB PDF)

Table S3 Mixtures Used in IL-7 Primary Proliferation Assay (42 KB PDF)

### Accession Numbers

The GenBank (http://www.ncbi.nlm.nih.gov/) accession numbers of the proteins discussed in this paper are α_1_-AR (AAB60352), ARP (AAB60352), DEAD box protein (AAH40185), minor capsid protein L2 (AAA79427), pVII protein (AAP49207 and AAA92212), PX2A (AAD42947), and TLR9 (BAB19259).

## Abbreviations

α1-AR: α-1B adrenergic receptor
aa: amino acid(s)
AUC: area under the curve
CDR3: complementarity-determining region 3
CNS: central nervous system
CSF: cerebrospinal fluid
L-aa: L-amino acid(s)
MS: multiple sclerosis
NLS: nuclear localization signal
ORF: open reading frame
PHA: phytohemagglutinin
PS-SCL: positional scanning synthetic combinatorial peptide library
SI: stimulatory index
TCC: T cell clone
TCR: T cell receptor
TLMV: TT-like mini virus
TTV: Torque Teno virus
URR: upstream regulatory region
