# Supplementary material for: Recognition of Conserved Amino Acid Motifs of Common Viruses and Its Role in Autoimmunity
Source: PLoS Pathog. 2005 Dec 16;1(4):e41. doi: 10.1371/journal.ppat.0010041 (PMC1315278; doi:10.1371/journal.ppat.0010041)
Supplement: Supplementary file 1 [file ppat.0010041.sg001.pdf]

Fig. S1

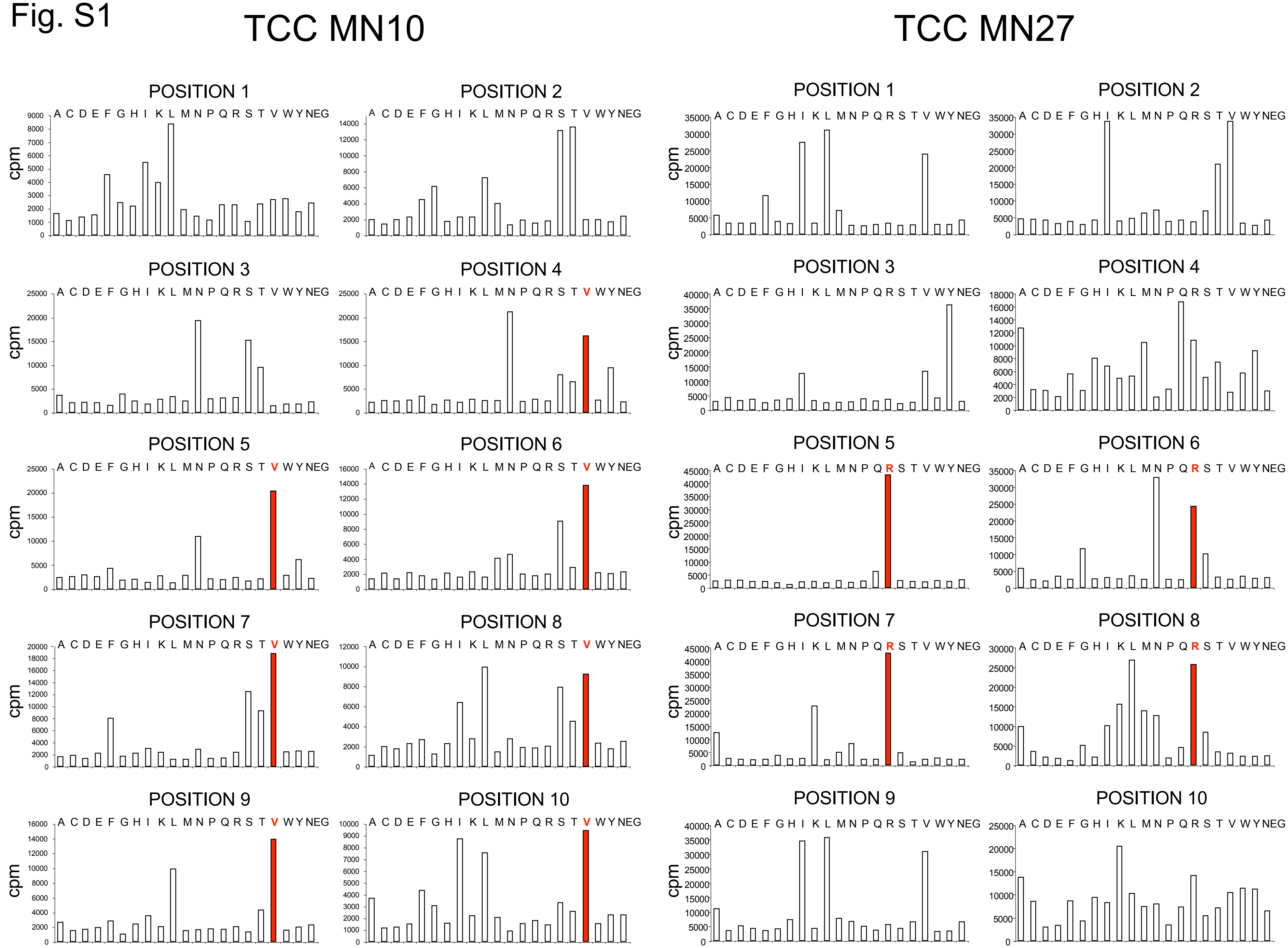
TCC MN10
TCC MN27
POSITION 1
POSITION 2
POSITION 3
POSITION 4
POSITION 5
POSITION 6
POSITION 7
POSITION 8
POSITION 9
POSITION 10
cpm
A C D E F G H I K L M N P Q R S T V W Y NEG

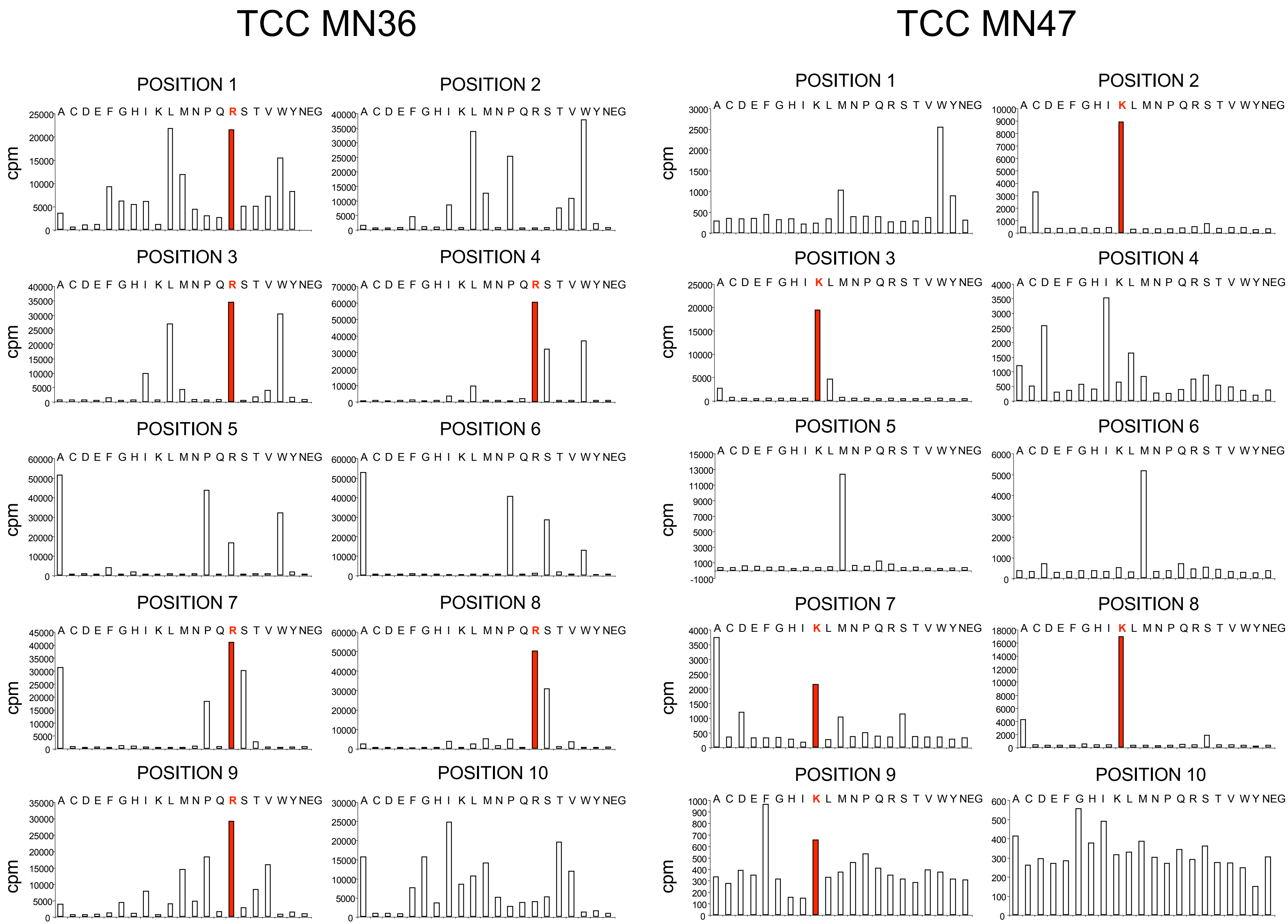
TCC MN36
TCC MN47
POSITION 1
POSITION 2
POSITION 3
POSITION 4
POSITION 5
POSITION 6
POSITION 7
POSITION 8
POSITION 9
POSITION 10
cpm
A C D E F G H I K L M N P Q R S T V W Y NEG
