# Supplementary material for: Recognition of Conserved Amino Acid Motifs of Common Viruses and Its Role in Autoimmunity
Source: PLoS Pathog. 2005 Dec 16;1(4):e41. doi: 10.1371/journal.ppat.0010041 (PMC1315278; doi:10.1371/journal.ppat.0010041)
Supplement: Supplementary file 2 [file ppat.0010041.sg002.pdf]

Fig. S2

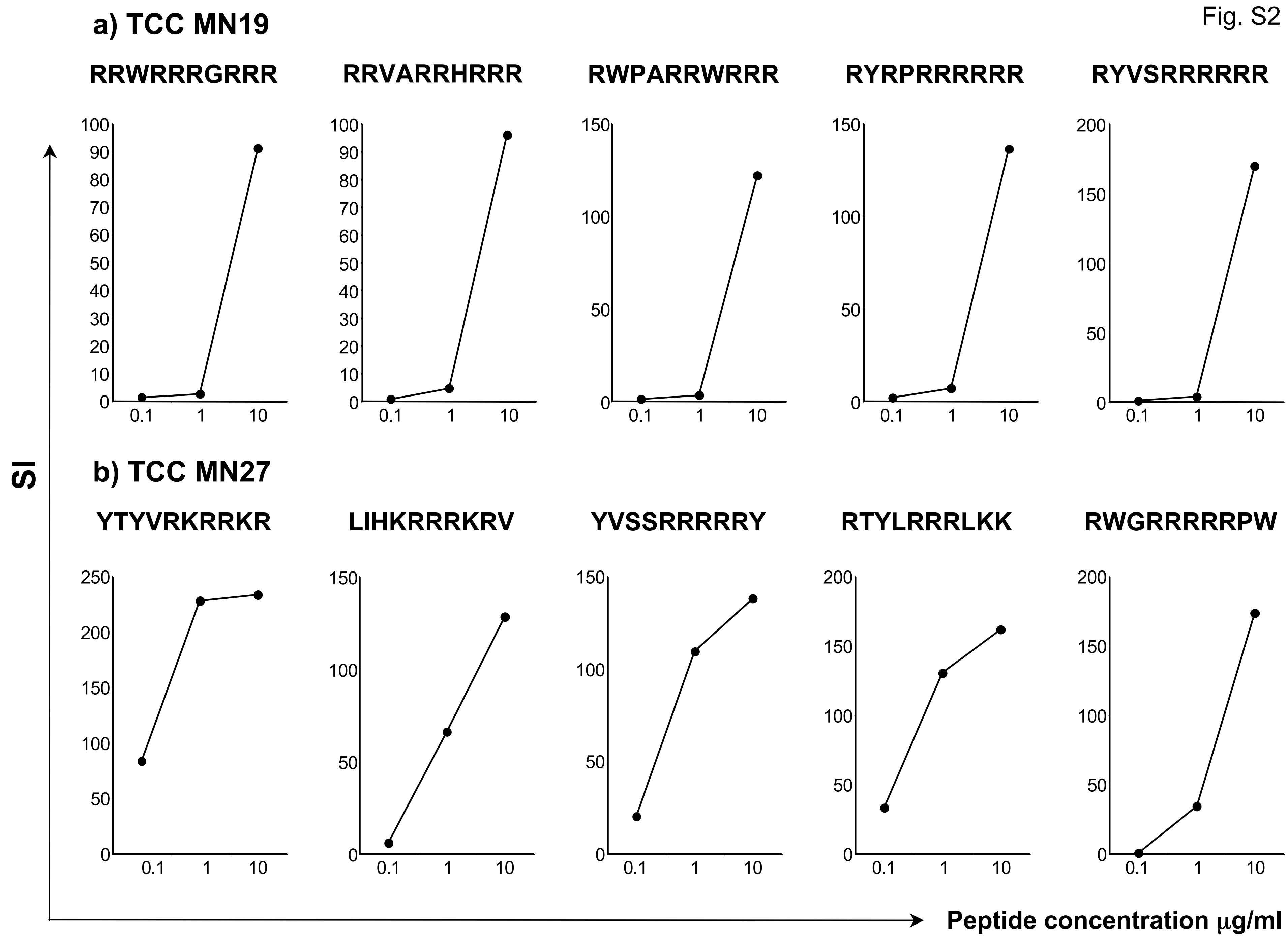

a) TCC MN19
RRWRRRGRRR
RRVARRHRRR
RWPARRWRRR
RYRPRRRRRR
RYVSRRRRRR
b) TCC MN27
YTYVRKRRKR
LIHKRRRKRV
YVSSRRRRRY
RTYLRRRLKK
RWGRRRRRPW
SI
Peptide concentration μg/ml
0.1
1
10
