# Supplementary material for: Recognition of Conserved Amino Acid Motifs of Common Viruses and Its Role in Autoimmunity
Source: PLoS Pathog. 2005 Dec 16;1(4):e41. doi: 10.1371/journal.ppat.0010041 (PMC1315278; doi:10.1371/journal.ppat.0010041)
Supplement: Supplementary file 3 [file ppat.0010041.st001.pdf]

Table SI. TTV clones

Sample	Templihi [a]				Genomiphi [a]			
	133/352 [b]	***249/351***	***472/352*** [b]	***473/351***	***133/352***	***249/351***	***472/352***	***473/351***
Serum month 0	12/15 pos -<br>TUS01 11x<br>JT34F 1x	9/11 pos -<br>TUS01 7x<br>TCHN-B 1x<br>JA2B 1x	9/9 pos -<br>JT34F 3x<br>sanIR1031<br>1x	11/11 pos -<br>JT34F 8x<br>sanIR1031 3x	6/8 pos -<br>TUS01 5x<br>TCHN-B 1x	6/18 pos -<br>TUS01 3x<br>TCHN-F 1x<br>TCHN-D 1x	10/10 pos -<br>JT34F 8x<br>sanIR1031 2x	11/11 pos -<br>JT34F 8x<br>sanIR1031 3x
Serum month 1	4/6 pos -<br>TUS01 4x	12/12 pos -<br>TUS01 8x<br>TCHN-F 2x<br>JA2B 1x<br>TCHN-B 1x	10/10 pos -<br>JT34F 6x<br>sanIR1031<br>4x	12/12 pos –<br>JT34F 6x<br>sanIR1031 5x<br>JA2B 1x	*navog*	6/9 pos –<br>TCHN-B 4x<br>TCHN-F 1x<br>JA2B 1x	8/8 pos –<br>sanIR1031 6x<br>JT34F 2x	12/12 pos –<br>JT34F 6x<br>JA2B 1x<br>sanIR1031 5x
Serum month 3	*navog*	*navog*	0/9 pos [c]	0/6 pos	*navog*	*navog*	0/10 pos	0/6 pos
Serum month 8	*navog*	*navog*	0/29	*navog*	*navog*	*navog*	0/8 pos	*navog*
Serum month 14	*navog*	*navog*	*navog*	*navog*	*navog*	*navog*	*navog*	*navog*
Serum month 24	*navog*	*navog*	*navog*	*navog*	*navog*	*navog*	*navog*	2/8 pos –<br>JT34F 1x<br>CT34F 1x
Brain patient 1	*navog*	*navog*	*navog*	9/11 pos –<br>JA2B 6x<br>JT34F 1x<br>sanIR1031 2x	*navog*	*navog*	*navog*	*navog*
Brain patient 3	*navog*	0/6 pos	*navog*	8/11 pos –<br>sanKC186 3x<br>T3PB 2x<br>yonKC-197<br>3x	*navog*	*navog*	*navog*	*navog*
Brain patient 6	*navog*	*navog*	*navog*	4/10 pos –<br>sanIR1031 2x<br>JT34F 1x<br>JA2B 1x	*navog*	*navog*	*navog*	*navog*
Brain patient 8	*navog*	*navog*	*navog*	6/11 pos –<br>JT34F 4x<br>JA2B 1x<br>sanIR1031 1x	*navog*	*navog*	*navog*	*navog*
Brain patient 9	*navog*	*navog*	*navog*	4/5 pos –<br>JT34F 4x	*navog*	*navog*	*navog*	*navog*
Brain patient 13	*navog*	0/10	*navog*	6/10 pos –<br>JA2B 3x<br>JT34F 1x<br>sanIR1031 2x	*navog*	*navog*	*navog*	*navog*

[a] – templiphi and genomiphi were used to amplify circular or total DNA from each sample prior to PCR amplification
[b] – primers used in each reaction. 133/352 were used in the first round and 249/351 for nested reaction. Similarly primers 472/352 were used in the initial PCR and 473/351 for the semi-nested reaction.
[c] – amplicons were visible on the gel and cloned, but none of the inserts harbored TTV sequences.
navog - no amplicon visible on gel
sanIR1031 – TTVsanIR1031
sanKC186 – TTVsanKC-186
