# Supplementary material for: Recognition of Conserved Amino Acid Motifs of Common Viruses and Its Role in Autoimmunity
Source: PLoS Pathog. 2005 Dec 16;1(4):e41. doi: 10.1371/journal.ppat.0010041 (PMC1315278; doi:10.1371/journal.ppat.0010041)
Supplement: Supplementary file 4 [file ppat.0010041.st002.pdf]

Table SII. Detailed information about all stimulatory peptides from TTV and TLMV

TT VIRUS ISOLATE	Accession #*	Protein	Peptide ID	OTHER ISOLATES	Sequence	Residues	SI MN19	SI MN27	SI MN36
TCHN-E	AF345522	ORF1	**MSI 148 130**	**2**	PWWRRRRRRR	(10 - 19)			
TCHN-E	AF345522	ORF1	**MSI 148 108**	**2**	WRRRRRRRWR	(12 - 21)			
TCHN-E	AF345522	ORF1	**MSI-144 126**	**3**	RRRRRRWRRR	(14 - 23)			
TCHN-E	AF345522	ORF1	MSI 144 144	2	RRRWRRRRRR	(17 - 26)			
TCHN-E	AF345522	ORF1	**MSI 148 107**	**2**	RWRRRRRRPR	(19 - 28)			
TCHN-E	AF345522	ORF1	**MSI 144 55**	**6**	RRRRRPRRRR	(22 - 31)			
TCHN-E	AF345522	ORF1	**MSI 148 134**	**2**	RRPRRRRPYR	(25 - 34)			
TCHN-E	AF345522	ORF1	**MSI 147 59**	**2**	PYRRRRPRRV	(32 - 41)			
TCHN-E	AF345522	ORF1	**MSI 148 102**	**6**	RVRRRRGRWR	(40 - 49)			
TCHN-E	AF345522	ORF1	**MSI 148 117**	**2**	RWGRRRRRRR	(54 - 63)			
TTV-like virus DXL1	AF315076	ORF1	MSI 148 130	2	PWWRRRRRRR	(10 - 19)			
TTV-like virus DXL1	AF315076	ORF1	MSI 148 108	2	WRRRRRRRWR	(12 - 21)			
TTV-like virus DXL1	AF315076	ORF1	MSI-144 126	3	RRRRRRWRRR	(13 - 22)			
TTV-like virus DXL1	AF315076	ORF1	MSI 144 144	2	RRRWRRRRRR	(17 - 26)			
TTV-like virus DXL1	AF315076	ORF1	MSI 148 107	2	RWRRRRRRPR	(19 - 28)			
TTV-like virus DXL1	AF315076	ORF1	MSI 144 55	6	RRRRRPRRRR	(22 - 31)			
TTV-like virus DXL1	AF315076	ORF1	MSI 148 134	2	RRPRRRRPYR	(25 - 34)			
TTV-like virus DXL1	AF315076	ORF1	MSI 147 59	2	PYRRRRPRRV	(32 - 41)			
TTV-like virus DXL1	AF315076	ORF1	MSI 148 102	6	RVRRRRGRWR	(40 - 49)			
TTV-like virus DXL1	AF315076	ORF1	MSI 148 117	2	RWGRRRRRRR	(54 - 63)			
CT30F	AB064597	ORF1	**MSI-144 12**		**RWRRRWRRRR**	(7 - 16)			
CT30F	AB064597	ORF1	**MSI 144 27**		**RLRRRRARRA**	(21 - 30)			
CT30F	AB064612	ORF1	**MSI 144 74**		**YVSSRRRRRY**	(40 - 49)			
CT30F	AB064597	ORF1	**MSI 144 21**		**RWRRPYRRRR**	(43 - 52)			
CT30F	AB064597	ORF1	**MSI 144 99**		**RPYRRRRRRG**	(46 - 55)			
CT30F	AB064597	ORF1	**MSI 144 184**		**RRGRRRRRRR**	(53 - 62)			
CT30F	AB064597	ORF1	**MSI 144 188**		**RGRRRRRRRR**	(54 - 63)			
CT30F	AB064597	ORF1	**MSI 144 188**		**RGRRRRRRRR**	(55 - 64)			
Kt-08F	AB054647	ORF1	**MSI 147 83**		**RRWRRRRRAW**	(15 - 24)			
Kt-08F	AB054647	ORF1	**MSI 147 36**		**RTYRRRRPLR**	(18 - 27)			
Kt-08F	AB054647	ORF1	**MSI 147 51**		**TYRRRRPLRW**	(19 - 28)			
Kt-08F	AB054647	ORF1	**MSI-144 113**		**RRYIRRRRLR**	(55 - 64)			
Kt-08F	AB054647	ORF1	**MSI 147 92**		**RYIRRRRLRR**	(56 - 65)			
Kt-08F	AB054647	ORF1	**MSI 147 82**		**YIRRRRLRRR**	(57 - 66)			
Pt-TTV6	AB041957	ORF1	**MSI 148 105**		**RWRRRRPRWR**	(11 - 20)			
Pt-TTV6	AB041957	ORF1	**MSI 144 151**		**RPRWRRWRRR**	(16 - 25)			
Pt-TTV6	AB041957	ORF1	**MSI-144 10**		**RWRRWRRRRT**	(18 - 27)			
Pt-TTV6	AB041957	ORF1	**MSI 147 65**		**RTWRRRPRRA**	(26 - 35)			
Pt-TTV6	AB041957	ORF1	**MSI 144 15**		**RPRRAVRRRR**	(31 - 40)			
Pt-TTV6	AB041957	ORF1	**MSI 147 95**		**RRVRRRGRGW**	(39 - 48)			
TCHN-G1	AF345521	ORF1	**MSI 144 5**		**RWRRWPRRRW**	(6 - 15)			
TCHN-G1	AF345521	ORF1	**MSI-144 128**	**13**	RRRWRRWRRR	(12 - 21)			
TCHN-G1	AF345521	ORF1	**MSI 144 23**	**7**	RWRRWRRRRR	(14 - 23)			
TCHN-G1	AF345521	ORF1	**MSI 144 154**	**7**	RRWRRRRRRR	(16 - 25)			
TCHN-G1	AF345521	ORF1	**MSI 144 18**		**RWRRRRRRRL**	(17 - 26)			
TCHN-G1	AF345521	ORF1	**MSI 147 71**		**RRRRRRPRRT**	(21 - 30)			
At-TTV3 Douroucouli	AB041961	ORF1	**MSI 144 132**	**2**	RRRYRRWRRR	(17 - 26)			
At-TTV3 Douroucouli	AB041961	ORF1	**MSI 144 145**		**RYRRWRRRRR**	(19 - 28)			
At-TTV3 Douroucouli	AB041961	ORF1	**MSI 144 179**		**RRWRRRRRWR**	(21 - 30)			
At-TTV3 Douroucouli	AB041961	ORF1	**MSI 147 87**		**RRRRRRTLVV**	(24 - 33)			
At-TTV3 Douroucouli	AB041961	ORF1	**MSI 144 6**		**RWRRRPRRRR**	(28 - 37)			

CH65-1	AB037926	ORF1	**MSI 147 54**		RRWRRRPRRW	(7 - 16)
CH65-1	AB037926	ORF1	**MSI-144 13**		RWRRWGRRRT	(15 - 24)
CH65-1	AB037926	ORF1	**MSI 144 166**		RRRFRRRRAR	(61 - 70)
CH65-1	AB037926	ORF1	**MSI 144 53**		FRRRRARRRK	(64 - 73)
CH65-1	AB037926	ORF1	**MSI-144 1**		RKRRAARRRT	(714-723)
CT23F	AB064595	ORF1	**MSI 147 80**		RRWRRRRRVW	(15 - 24)
CT23F	AB064595	ORF1	**MSI-144 115**		AVRRRRRGRY	(31 - 40)
CT23F	AB064595	ORF1	**MSI 144 88**		YVSKRRRFRR	(40 - 49)
CT23F	AB064595	ORF1	**MSI 144 167**		RRRFRRRRRR	(44 - 53)
CT23F	AB064595	ORF1	**MSI 144 178**		RRRRRRGRPR	(49 - 58)
CT25F	AB064596	ORF1	**MSI 147 94**		GRWRRRRRVW	(15 - 24)
CT25F	AB064596	ORF1	**MSI 144 86**		YVSRRRRYRR	(40 - 49)
CT25F	AB064596	ORF1	**MSI 144 173**		RRYRRRLRRR	(45 - 54)
CT25F	AB064596	ORF1	**MSI 144 180**		RRLRRRGRRR	(49 - 58)
CT25F	AB064596	ORF1	**MSI 144 25**		RLRRRGRRRY	(50 - 59)
JaCHC2c	AB030486	ORF1	**MSI 144 148**		RRRSRRWRRR	(7 - 16)
JaCHC2c	AB030486	ORF1	**MSI-144 128**	**13**	RRRWRRWRRR	(15 - 24)
JaCHC2c	AB030486	ORF1	**MSI 144 23**	**7**	RWRRWRRRRR	(17 - 26)
JaCHC2c	AB030486	ORF1	**MSI 144 55**	**6**	RRRRRPRRRR	(22 - 31)
JaCHC2c	AB030486	ORF1	**MSI 144 181**		RRPARRCRRR	(30 - 39)
Mf-TTV3 Japaese Macaque	AB041958	ORF1	**MSI 144 28**		WWRRWPRRRR	(4 - 13)
Mf-TTV3	AB041958	ORF1	**MSI 144 7**		RWRRWGRRRV	(14 - 23)
Mf-TTV3	AB041958	ORF1	**MSI 144 162**		RRVRRRRRRR	(39 - 48)
Mf-TTV3	AB041958	ORF1	**MSI 144 49**		RVRRRRRRRV	(40 - 49)
Mf-TTV3	AB041958	ORF1	**MSI 147 81**		RLRRRRRLRR	(57 - 66)
SANBAN	AB025946	ORF1	**MSI 144 20**	**3**	RWRRWPARRW	(8 - 17)
SANBAN	AB025946	ORF1	**MSI 144 161**	**4**	RWPARRWRRR	(11 - 20)
SANBAN	AB025946	ORF1	**MSI 144 154**	**7**	RRWRRRRRRR	(15 - 24)
SANBAN	AB025946	ORF1	**MSI 144 31**	**5**	RWRRRRRRRP	(16 - 25)
SANBAN	AB025946	ORF1	**MSI 147 56**		RRRRRRPLRR	(18 - 27)
TTVsan-S039	AB038620	ORF1	**MSI 144 20**	**3**	RWRRWPARRW	(8 - 17)
TTVsan-S039	AB038620	ORF1	**MSI 144 161**	**4**	RWPARRWRRR	(11 - 20)
TTVsan-S039	AB038620	ORF1	**MSI 144 154**	**7**	RRWRRRRRRR	(15 - 24)
TTVsan-S039	AB038620	ORF1	**MSI 144 31**	**5**	RWRRRRRRRP	(16 - 25)
TTVsan-S039	AB038620	ORF1	**MSI 144 163**	**3**	RRPARRYRRR	(32 - 41)
CT43F	AB064598	ORF1	**MSI 144 19**		RPWRPWRRRG	(9 - 18)
CT43F	AB064614	ORF1	**MSI 144 65**		YVSRRRRRRR	(40 - 49)
CT43F	AB064598	ORF1	**MSI 144 191**		RRRYRRGRRR	(48 - 57)
CT43F	AB064598	ORF4	**MSI 147 77**		RKPRRRRLLF	(118 - 127)
HEL32/6a	AY034068	ORF1	**MSI 144 142**	**2**	RRRWRRRRAR	(19 - 28)
HEL32/6a	AY034068	ORF1	**MSI 144 26**		WRRRRARRRG	(22 - 31)
HEL32/6a	AY034068	ORF1	**MSI 148 102**	**6**	RVRRRRGRWR	(41 - 50)
HEL32/6a	AY034068	ORF1	**MSI 144 132**	**2**	RRRYRRWRRR	(50 - 59)
JT05F	AB064600	ORF1	**MSI 147 48**		AIRRRRPRRT	(20 - 29)
JT05F	AB064600	ORF1	**MSI 144 170**	**3**	RRRYRRRRRR	(44 - 53)
JT05F	AB064600	ORF1	**MSI-144 100**		RRYRRRRRRT	(45 - 54)
JT05F	AB064600	ORF1	**MSI-144 129**		RYRRRRRRTR	(46 - 55)
JT14F	AB064601	ORF1	**MSI 147 91**		RPWRRRRRPW	(10 - 19)
JT14F	AB064601	ORF1	**MSI-144 122**		FVRRRPRRRY	(29 - 38)
JT14F	AB064601	ORF1	**MSI 144 185**		RWRRRRYRRR	(41 - 50)
JT14F	AB064601	ORF1	**MSI 144 48**		RLRRGRRRRG	(50 - 59)

JT41F	AB064603	ORF1	**MSI 147 96**		**RWGRRRRRPW**	(12 - 21)
JT41F	AB064603	ORF1	**MSI 144 192**	**4**	**RYVSRRRRRR**	(39 - 48)
JT41F	AB064603	ORF1	**MSI 144 69**	**5**	**YVSRRRRRRY**	(40 - 49)
JT41F	AB064603	ORF1	**MSI 144 71**	**4**	**RRYYRRRLRR**	(47 - 56)
L03	AF371370	ORF1	**MSI 144 72**		**AVPRRRRRRR**	(32 - 41)
L03	AF371370	ORF1	**MSI 144 45**		**RWGRRRPRRR**	(46 - 55)
L03	AF371370	ORF3	**MSI-144 114**		**RVYYKRRRRK**	(55 - 64)
L03	AF371370	ORF1	**MSI 144 94**		**VYYKRRRRKT**	(56 - 65)
SENV-H	AY183662	ORF1	**MSI 144 43**		**RPRRWRRRRR**	(13 - 22)
SENV-H	AY183662	ORF1	MSI 144 154	7	**RRWRRRRRRR**	(15 - 24)
SENV-H	AY183662	ORF1	**MSI 144 9**		**RWRRRRRRRV**	(16 - 25)
SENV-H	AY183662	ORF1	**MSI 144 17**		**RLRRRRRRRK**	(60 - 69)
TTVsan-IR1031	AB038619	ORF1	**MSI 144 20**	**3**	**RWRRWPARRW**	(8 - 17)
TTVsan-IR1031	AB038619	ORF1	**MSI 144 161**	**4**	**RWPARRWRRR**	(11 - 20)
TTVsan-IR1031	AB038619	ORF1	**MSI 144 154**	**7**	**RRWRRRRRRR**	(15 - 24)
TTVsan-IR1031	AB038619	ORF1	**MSI 144 31**	**5**	**RWRRRRRRRP**	(16 - 25)
TTV CH71 Pan troglodytes	AB049608	ORF1	**MSI 144 174**		**RRRRARWRRR**	(8 - 17)
TTV CH71 Pan troglodytes	AB049608	ORF1	**MSI 144 36**		**WRRRAAARRW**	(14 - 23)
TTV CH71 Pan troglodytes	AB049608	ORF1	**MSI 144 164**		**RAAARRWRRR**	(17 - 26)
TTV CH71 Pan troglodytes	AB049608	ORF1	**MSI 148 102**	**6**	**RVRRRRGRWR**	(42 - 51)
TUS01	AB017613	ORF1	**MSI-144 128**	**13**	**RRRWRRWRRR**	(13 - 22)
TUS01	AB017613	ORF1	**MSI 144 23**	**7**	**RWRRWRRRRR**	(15 - 24)
TUS01	AB017613	ORF1	**MSI 144 168**		**RRWRRRRRRL**	(17 - 26)
TUS01	AB017613	ORF1	**MSI-144 111**		**RVRRRRRTRR**	(45 - 54)
TX011(A)	AB011492	ORF1	**MSI 144 30**		**RIRRPRRRRA**	(21 - 30)
TX011(A)	AB011492	ORF1	**MSI 144 51**		**RRRRAAYRRR**	(26 - 35)
TX011(A)	AB011492	ORF1	**MSI-144 123**		**AAYRRRRTRR**	(30 - 39)
TX011(A)	AB011492	ORF1	**MSI 148 102**	**6**	**RVRRRRGRWR**	(40 - 49)
US35	AF122920	ORF1	**MSI 144 61**		**RRRRPWRRRR**	(9 - 18)
US35	AF122920	ORF1	**MSI-144 128**	**13**	**RRRWRRWRRR**	(16 - 25)
US35	AF122920	ORF1	**MSI 144 23**	**7**	**RWRRWRRRRR**	(18 - 27)
US35	AF122920	ORF1	**MSI 144 55**	**6**	**RRRRRPRRRR**	(23 - 32)
yon-KC009	AB038621	ORF1	**MSI 147 68**	**3**	**RVRRRRPRRT**	(22 - 31)
yon-KC009	AB038621	ORF1	**MSI 144 192**	**4**	**RYVSRRRRRR**	(39 - 48)
yon-KC009	AB038621	ORF1	**MSI 144 69**	**5**	**YVSRRRRRRY**	(40 - 49)
yon-KC009	AB038621	ORF1	**MSI 144 71**	**4**	**RRYYRRRLRR**	(47 - 56)
yon-KC197	AB038624	ORF1	**MSI 147 68**	**3**	**RVRRRRPRRT**	(22 - 31)
yon-KC197	AB038624	ORF1	**MSI 144 192**	**4**	**RYVSRRRRRR**	(39 - 48)
yon-KC197	AB038624	ORF1	**MSI 144 69**	**5**	**YVSRRRRRRY**	(40 - 49)
yon-KC197	AB038624	ORF1	**MSI 144 71**	**4**	**RRYYRRRLRR**	(47 - 56)
yon-LC011	AB038622	ORF1	**MSI 147 68**	**3**	**RVRRRRPRRT**	(22 - 31)
yon-LC011	AB038622	ORF1	**MSI 144 192**	**4**	**RYVSRRRRRR**	(39 - 48)
yon-LC011	AB038622	ORF1	**MSI 144 69**	**5**	**YVSRRRRRRY**	(40 - 49)
yon-LC011	AB038622	ORF1	**MSI 144 71**	**4**	**RRYYRRRLRR**	(47 - 56)
G97801	AB011486	ORF1	**MSI 144 158**	**8**	**RRRRRRWRRW**	(7 - 16)
G97801	AB011486	ORF1	**MSI-144 128**	**13**	**RRRWRRWRRR**	(10 - 19)
G97801	AB011486	ORF1	**MSI 144 3**	**18**	**RRRRPARRRG**	(28 - 37)
GH1	AF122913	ORF1	**MSI 144 158**	**8**	**RRRRRRWRRW**	(7 - 16)
GH1	AF122913	ORF1	**MSI-144 128**	**13**	**RRRWRRWRRR**	(10 - 19)

GH1	AF122913	ORF1	**MSI 144 3**	**18**	RRRRPARRRG	(28 - 37)
JA1	AF122916	ORF1	**MSI-144 128**	**13**	RRRWRRWRRR	(16 - 25)
JA1	AF122916	ORF1	**MSI 144 23**	**7**	RWRRWRRRRR	(18 - 27)
JA1	AF122916	ORF1	**MSI 144 55**	**6**	RRRRRPRRRR	(23 - 32)
JA20	AF122914	ORF1	**MSI 144 158**	**8**	RRRRRRWRRW	(7 - 16)
JA20	AF122914	ORF1	**MSI-144 128**	**13**	RRRWRRWRRR	(10 - 19)
JA20	AF122914	ORF1	**MSI 144 3**	**18**	RRRRPARRRG	(28 - 37)
JA2B	AF122918	ORF1	**MSI-144 128**	**13**	RRRWRRWRRR	(16 - 25)
JA2B	AF122918	ORF1	**MSI 144 23**	**7**	RWRRWRRRRR	(18 - 27)
JA2B	AF122918	ORF1	**MSI 144 55**	**6**	RRRRRPRRRR	(23 - 32)
JT03F	AB064599	ORF1	**MSI 144 46**		**RPWRRWRRRR**	(9 - 18)
JT03F	AB064599	ORF1	**MSI-144 130**	**2**	RYRRRRRRGR	(46 - 55)
JT03F	AB064599	ORF1	**MSI 144 170**	**3**	RRRYRRRRRR	(44 - 53)
Kt-10F	AB054648	ORF1	**MSI 147 75**		**RTRRRRRLPV**	(18 - 27)
Kt-10F	AB054648	ORF1	**MSI 147 44**		**TRYARRRRVR**	(34 - 43)
Kt-10F	AB054648	ORF1	**MSI 147 76**		**RLRRRRRRGW**	(44 - 53)
L01	AY026465	ORF1	**MSI 144 4**		**RWPRPARRRP**	(26 - 35)
L01	AY026465	ORF1	**MSI 147 70**		**RPARRRPRRR**	(29 - 38)
L01	AY026465	ORF1	**MSI-144 116**	**2**	RVRRRRRWRR	(39 - 48)
Mf-TTV9 Japaese Macaque	AB041959	ORF1	**MSI 144 14**		**RWRRWRRRRG**	(8 - 17)
Mf-TTV9	AB041959	ORF1	**MSI 144 33**		**RTARRARRRR**	(30 - 39)
Mf-TTV9	AB041959	ORF1	**MSI 144 78**		**AVRRRRRRRV**	(41 - 50)
PMV	AF261761	ORF1	**MSI 144 42**		**RRRRWPRRRR**	(9 - 18)
PMV	AF261761	ORF1	**MSI 144 70**		**RTYIRRRRRR**	(55 - 64)
PMV	AF261761	ORF1	**MSI 144 68**		**YIRRRRRRKK**	(57 - 66)
SAa-01	AB060597	ORF1	**MSI 147 52**		**RLRRRRPRRP**	(23 - 32)
SAa-01	AB060597	ORF1	**MSI 144 41**	**2**	RPRRPVRRRR	(28 - 37)
SAa-01	AB060597	ORF1	**MSI-144 118**		**AVRRRRRPKK**	(60 - 69)
SAj-30	AB060595	ORF1	**MSI 144 149**		**RGRWRRWRRR**	(15 - 24)
SAj-30	AB060595	ORF1	**MSI 144 23**	**7**	RWRRWRRRRR	(17 - 26)
SAj-30	AB060595	ORF1	**MSI 147 47**		**RIPRRRPRRP**	(26 - 35)
SENV-D	AY072045	ORF1	MSI 144 81	2	RTYGRRRRRR	(36 - 45)
SENV-D	AY072045	ORF1	MSI 144 82	2	RRYIRRRRRK	(63 - 72)
SENV-D	AY072045	ORF1	MSI 144 73	2	YIRRRRRKKK	(65 - 74)
TA278	AB008394	ORF1	**MSI 144 158**	**8**	RRRRRRWRRW	(7 - 16)
TA278	AB008394	ORF1	**MSI-144 128**	**13**	RRRWRRWRRR	(22 - 31)
TA278	AB008394	ORF1	**MSI 144 3**	**18**	RRRRPARRRG	(28 - 37)
TCHN-C2	AF345527	ORF1	**MSI 144 142**	**2**	RRRWRRRRAR	(17 - 26)
TCHN-C2	AF345527	ORF1	**MSI 144 60**		**RPRRLPRRRY**	(25 - 34)
TCHN-C2	AF345527	ORF1	**MSI 144 156**		**RYRGRRVRRR**	(40 - 49)
TCHN-G2	AF345529	ORF1	**MSI 144 154**	**7**	RRWRRRRRRR	(15 - 24)
TCHN-G2	AF345529	ORF1	**MSI 144 31**	**5**	RWRRRRRRRP	(16 - 25)
TCHN-G2	AF345529	ORF1	**MSI 144 163**	**2**	RRPARRYRRR	(32 - 41)
TJN01	AB028668	ORF1	**MSI 144 81**	**2**	RTYGRRRRRR	(36 - 45)
TJN01	AB028668	ORF1	**MSI 144 82**	**2**	RRYIRRRRRK	(63 - 72)
TJN01	AB028668	ORF1	**MSI 144 73**	**2**	YIRRRRRKKK	(65 - 74)

TJN02	AB028669	ORF1	**MSI 144 161**	**4**	RWPARRWRRR	(11 - 20)
TJN02	AB028669	ORF1	**MSI 144 154**	**7**	RRWRRRRRRR	(15 - 24)
TJN02	AB028669	ORF1	**MSI 144 31**	**5**	RWRRRRRRRP	(16 - 25)
TRM1	AB026345	ORF1	**MSI 144 158**	**8**	RRRRRRWRRW	(7 - 16)
TRM1	AB026345	ORF1	**MSI-144 128**	**13**	RRRWRRWRRR	(10 - 19)
TRM1	AB026345	ORF1	**MSI 144 3**	**18**	RRRRPARRRG	(28 - 37)
TTV021	AF254410	ORF1	**MSI 147 29**		**RVYLRRRRRR**	(15 - 24)
TTV021	AF254410	ORF1	**MSI 147 73**		**RRRRRRKRLV**	(21 - 30)
TTV021	AF254410	ORF1	**MSI-144 62**		**RVYLRRRRRR**	(39 - 48)
TTVCHN2	AF129887	ORF1	**MSI 147 88**		**GLWRRRRRRW**	(4 - 13)
TTVCHN2	AF129887	ORF1	**MSI 144 136**		**RWRRRRWRRR**	(15 - 24)
TTVCHN2	AF129887	ORF1	**MSI 144 155**		**RRRRRRGRWR**	(43 - 52)
VT416 clone="pTZVT416"	AB041007	ORF1	**MSI 144 158**	**8**	RRRRRRWRRW	(7 - 16)
VT416	AB041007	ORF1	**MSI-144 128**	**13**	RRRWRRWRRR	(10 - 19)
VT416	AB041007	ORF1	**MSI 144 3**	**18**	RRRRPARRRG	(28 - 37)
CT39F	AB064604	ORF1	**MSI 144 32**	**2**	RWPRRSRRRP	(26 - 35)
CT39F	AB064604	ORF1	**MSI-144 116**	**2**	RVRRRRRWRR	(39 - 48)
JA9	AF122915	ORF1	**MSI 144 158**	**8**	RRRRRRWRRW	(7 - 16)
JA9	AF122915	ORF1	**MSI-144 128**	**13**	RRRWRRWRRR	(10 - 19)
JT19F	AB064602	ORF1	**MSI 144 170**	**3**	RRRYRRRRRR	(44 - 53)
JT19F	AB064602	ORF1	**MSI-144 130**	**2**	RYRRRRRRGR	(46 - 55)
JT33F	AB064606	ORF1	**MSI 144 16**		**RWPRSARRRP**	(26 - 35)
JT33F	AB064606	ORF1	**MSI 147 67**		**RSARRRPRRR**	(29 - 38)
KAV	AF435014	ORF1	**MSI 144 157**		**RYRPRRRRRR**	(53 - 62)
KAV	AF435014	ORF1	**MSI 144 54**		**RPRRRRRRRK**	(55 - 64)
KC200/1-12G (SAMBAN)	AB024380	ORF1	**MSI-144 127**		**RRRWRRWRAR**	(19 - 28)
KC200/1-12G (SAMBAN)	AB024380	ORF1	**MSI 144 29**		**AGRRRARRRV**	(33 - 42)
P/1C1	AF298585	ORF1	**MSI 144 133**		**RRRWRRWRTR**	(9 - 18)
P/1C1	AF298585	ORF1	**MSI 148 102**	**6**	RVRRRRGRWR	(41 - 50)
SAa-10	AB060594	ORF1	**MSI 144 172**		**RRWRRRGRRR**	(14 - 23)
SAa-10	AB060594	ORF1	**MSI 144 35**		**RWRRRGRRRR**	(15 - 24)
SAf-09	AB060596	ORF1	**MSI 144 169**	**2**	RRRRRRPRVR	(34 - 43)
SAf-09	AB060596	ORF4	**MSI 148 169**		**TVRARKRLRF**	(115 - 124)
So-TTV2 Cotton-top tamarin	AB041960	ORF3	**MSI 147 49**		**IRKKRRPGRG**	(10-19)
So-TTV2 Cotton-top tamarin	AB041960	ORF1	**MSI-144 126**	**3**	RRRRRRWRRR	(32 - 41)
TCHN-B	AF348409	ORF1	**MSI 144 41**	**2**	RPRRPVRRRR	(28 - 37)
TCHN-B	AF348409	ORF1	**MSI 144 169**	**2**	RRRRRRPRVR	(34 - 43)
TTV CH65-2	AB049607	ORF1	**MSI 144 24**		**RWRRWRRRRP**	(3 - 12)
TTV CH65-2	AB049607	ORF1	**MSI 147 84**		**RWGRRRGRLR**	(47 - 56)
TTVCHN1	AF079173	ORF1	**MSI 144 158**	**8**	RRRRRRWRRW	(7 - 16)
TTVCHN1	AF079173	ORF1	**MSI 144 138**		**RRRWRRWRPR**	(10 - 19)
TX011(O)	AB011493	ORF1	**MSI 144 183**		**RRRWRRWRRQ**	(10 - 19)
TX011(O)	AB011493	ORF1	**MSI 144 3**	**18**	RRRRPARRRG	(28 - 37)

TYM9	AB050448	ORF1	**MSI 148 101**		**LVRRRRKRYR**	(4 - 13)			
TYM9	AB050453	ORF1	**MSI 144 75**	**2**	RTYLRRRLKK	(27 - 36)			
yon-KC186	AB038623	ORF1	**MSI 147 55**		**RLRRRRPRRT**	(22 - 31)			
yon-KC186	AB038623	ORF1	**MSI 144 69**	**5**	YVSRRRRRRY	(40 - 49)			
BDH1 strain="BDH1"	AF116842	ORF1	**MSI 144 3**	**18**	RRRRPARRRG	(28 - 37)			
Cf-TTV10 "dog"	AB076002	ORF1	**MSI 148 104**		**GYARRRWRRR**	(22 - 31)			
CT44F	AB064605	ORF1	**MSI 144 32**	**2**	RWPRRSRRRP	(26 - 35)			
Fc-TTV4	AB076003	ORF1	**MSI-144 124**		**GIYRRWRRWR**	(33 - 42)			
G102001	AB011488	ORF1	**MSI 144 3**	**18**	RRRRPARRRG	(28 - 37)			
G103301	AB011487	ORF1	**MSI 144 3**	**18**	RRRRPARRRG	(28 - 37)			
G104901	AB011489	ORF1	**MSI 144 2**		**RRRRPARRRA**	(28 - 37)			
G105001	AB011490	ORF1	**MSI 144 3**	**18**	RRRRPARRRG	(28 - 37)			
G88801	AB011491	ORF1	**MSI 144 3**	**18**	RRRRPARRRG	(28 - 37)			
JA4	AF122917	ORF1	**MSI 144 3**	**18**	RRRRPARRRG	(28 - 37)			
JA10	AF122919	ORF1	**MSI 144 3**	**18**	RRRRPARRRG	(28 - 37)			
JaCHCTC19 clone="JaCHCTC	AB030487	ORF1	**MSI 144 3**	**18**	RRRRPARRRG	(28 - 37)			
KC008/2-3G (SAMBAN)	AB024360	ORF1	**MSI 147 69**	**2**	RTRRRRPRRR	(26 - 35)			
KC037/1-12G (SAMBAN)	AB024370	ORF1	**MSI 144 52**		**RRRRAAFRRR**	(26 - 35)			
L02	AY026466	ORF1	**MSI 147 69**	**2**	RTRRRRPRRR	(26 - 35)			
SAa-38	AB060593	ORF1	**MSI 144 97**		**RRYLRRRKRR**	(65 - 74)			
Saa-39	**BAB69900**	ORF1	**MSI 144 75**	**2**	RTYLRRRLKK	(64 - 73)			
Sd-TTV31 "pig"	AB076001	ORF1	**MSI 147 50**		**RIRRRRPWRR**	(27 - 36)			
TCHN-D1	AF345524	ORF1	**MSI 144 39**	**2**	RPRRRTRRRT	(30 - 39)			
TCHN-D2	AF345525	ORF1	**MSI 144 39**	**2**	RPRRRTRRRT	(30 - 39)			
TK16	AB026346	ORF1	**MSI 144 3**	**18**	RRRRPARRRG	(28 - 37)			
TP1-3	AB026347	ORF1	**MSI 144 3**	**18**	RRRRPARRRG	(28 - 37)			
TWH	AF351132	ORF1	**MSI-144 131**		**RKRWRRWRRR**	(10 - 19)			
TY96117	AB011494	ORF1	**MSI 144 3**	**18**	RRRRPARRRG	(28 - 37)			

**TTV-LIKE MINI VIRUS**	**Accession no**				**Sequence**	**Location**	**MN19**	**MN27**	**MN36**
TLMV-CBD231	AB026930	ORF1	**MSI 144 79**		**RIYYRERIRK**	(644 - 653)			
TLMV-CBD279	AB026931	ORF1	**MSI 147 86**		**LRFRRRRLRR**	(20 - 29)			

TLMV-CLC156	AB038627	ORF1	**MSI 148 123**	**YYPRRRRFWR**	(9 - 18)			
TLMV-CLC156	AB038627	ORF1	**MSI 148 125**	**RRFWRRRRYR**	(37 - 46)			
TLMV-NLC026	AB038630	ORF1	**MSI 148 163**	**PTYRRRRWRR**	(15 - 24)			
TLMV-NLC026	AB038630	ORF1	**MSI 148 98**	**TYRRRRWRRR**	(16 - 25)			
PB4TL	AF291073	ORF1	**MSI 144 101**	**NYYYRRRRRP**	(5 - 14)			
PB4TL	AF291073	ORF1	**MSI 144 117**	**YYYRRRRRPR**	(6 - 15)			

* GenBank Database

	**SI**
	SI < 3
	3 < SI < 10
	10 < SI < 50
	50 < SI < 100
	SI > 100
