# Supplementary material for: Recognition of Conserved Amino Acid Motifs of Common Viruses and Its Role in Autoimmunity
Source: PLoS Pathog. 2005 Dec 16;1(4):e41. doi: 10.1371/journal.ppat.0010041 (PMC1315278; doi:10.1371/journal.ppat.0010041)
Supplement: Supplementary file 5 [file ppat.0010041.st003.pdf]

Table SIII. Mixtures used in IL-7 primary proliferation assay

Mixture 1- Arginine enriched peptides

Peptide	Sequence	R	Organism
MSI 144 154	RRWRRRRRRR	9	TT virus
MSI 144 158	RRRRRRWRRW	8	TT virus
MSI 144 157	RYRPRRRRRR	8	TT virus
MSI 144 132	RRRYRRWRRR	8	TT virus
MSI 144 70	RTYIRRRRRR	7	TT virus
MSI 144 78	AVRRRRRRRV	7	TT virus
MSI 144 97	RRYLRRRKRR	7	TT virus
MSI 147 68	RVRRRRPRRT	7	TT virus
MSI 144 69	YVSRRRRRRY	6	TT virus
MSI 147 95	RRVRRRGRGW	6	TT virus

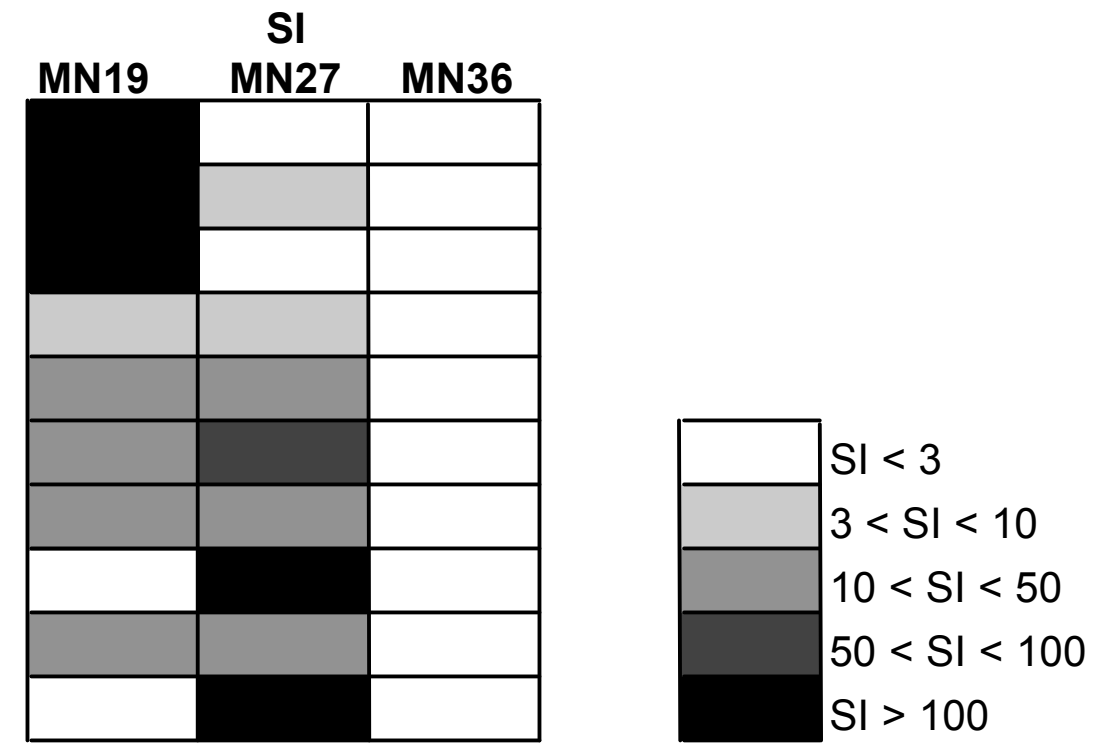

Mixture 2- Lysine enriched peptides

	Sequence	K	Organism
MSI 154 181	KKKKKTKKKK	9	Chimpanzee cytomegalovirus
MSI 154 180	NKKKKKTKKK	8	Chimpanzee cytomegalovirus
MSI 154 182	KKKNKLVKKK	7	Equine encephalitis virus
MSI 154 183	KKKKNPGKKK	7	Equine encephalitis virus
MSI 154 184	KKKKNQGKKK	7	Equine encephalitis virus
MSI 154 189	KKKVVTKKKK	7	Chlamydophila pneumoniae
MSI 154 154	KAKAKQQKKK	6	Equine encephalitis virus
MSI 154 159	HSKKKRSKKK	6	TT virus
MSI 154 166	KQKERKSKKK	6	TT virus
MSI 154 187	YKKKTPTKKK	6	Chlamydophila pneumoniae

Mixture 3- Peptides not enriched in any aa

	Sequence	Protein
MSI 126 62	ARLAAARLNR	Retina-specific guanylyl cyclase
MSI 126 80	IIRAAADRDR	Human chaperone
MSI 147 105	TMKLMMEKVD	Borna disease virus
MSI 147 5	FRKDMILKIF	Campylobacter jejuni
MSI 148 162	HVYVRARLPD	Pseudorabies virus
MSI 148 191	KILPRHRLIK	Treponema pallidum
MSI 95 3	AIRLPPGRLL	Human herpesvirus 5
MSI 95 55	AIRSAWWWWL	Teorethical peptide
MSI 95 7	SVRSSPRFLL	Influenza C virus
MSI 95 96	DWEHMKKPEA	Teorethical peptide

Mixture 4- Complex mixture of completely random peptides

N46-1	XXXXXXXXXX
